# CASE REPORT Playing Football Burns More Than Just Calories

**Published:** 2010-07-16

**Authors:** Richard A. J. Wain, Syed H. A. Shah

**Affiliations:** Department of Plastic & Reconstructive Surgery, Royal Preston Hospital, Preston, Lancashire, United Kingdom

## Abstract

**Objective:** To highlight the case of a sports-related alkali burn due to a common household chemical and emphasize the importance of a detailed medical history in chemical burns patients. **Methods:** A single-patient case study is presented along with references from existing literature. **Results:** Alkaline burn injuries associated with sports have previously been described in the literature; however, this case demonstrates an unusual presentation of a chemical burn with a readily available household substance. **Conclusion:** Chemical burns can present in atypical ways. Detailed history and thorough clinical examination is essential in determining the correct diagnosis and therefore implementing the most appropriate management plan.

Burn injuries associated with sports are well reported and often secondary to hydrated lime exposure, used for marking lines on football or rugby pitches.^1^,^2^ We present the case of an adult footballer who sustained full-thickness ankle burns after wearing protective shin guards that were inadvertently impregnated with the caustic alkaline agent sodium hydroxide (NaOH).

## CASE REPORT

A 27-year-old fit and healthy man presented to the plastic surgery unit several hours after finishing a game of football. He complained of pain and burning to both ankles, which had been gradually worsening during and after the match. The patient denied any exposure to thermal or chemical sources, and no similar injuries were sustained by other players. He was wearing his usual boots, long socks, protective shin guards, and shorts, and the pitch was not marked out using lime.

**Figure 1:**
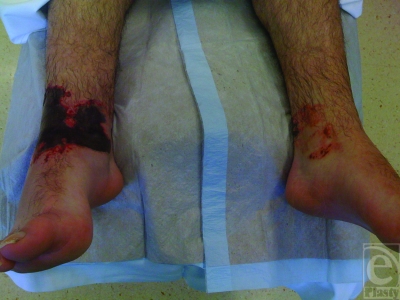
Anterior aspect of both lower legs demonstrating the burned area.

**Figure 2:**
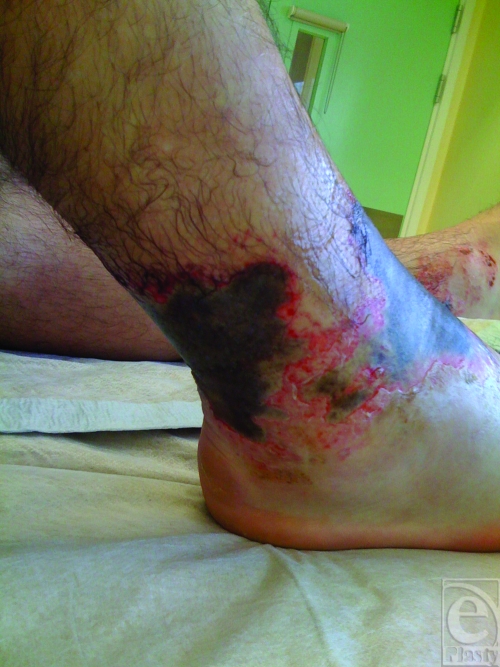
Lateral aspect of right lower leg demonstrating burns anteriorly and posteriorly.

On examination, there were areas of necrotic skin and erythema present on both ankles, with the right ankle being significantly worse than the left (Figs 1 and 2). The total body surface area involved was approximately 2.5%. There were no burns elsewhere, and the patient was systemically well.

The pattern of burn injury was unusual in that it was confined to the distal parts of the lower leg, in the ankle region. This area corresponded exactly to the area covered by the elasticated-cloth part of the patient's shin guards.

The patient could not attribute his injuries to any particular cause, and he did not remember anything out of the ordinary happening to him before the game. On more detailed questioning, however, it was discovered that the patient had been at work at a bar just prior to his football match. It transpired that the patient stored his football kit in the bar's basement, near to some cleaning products.

**Figure 3:**
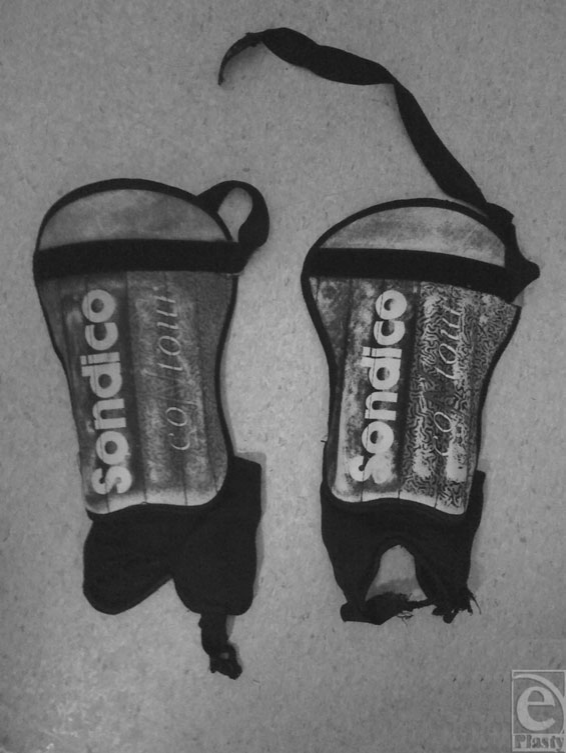
Protective shin guards worn by the patient showing the absorbent cloth section inferiorly which coincides with the burned region.

One of these cleaning products, containing sodium hydroxide, had leaked onto the kit bag and soaked into the absorbent cloth part of the patient's protective shin pads (Fig 3). It is interesting to note that the patient insists the shin guards were entirely dry prior to the match, implying that the chemical had time to dry in the material. The chemical then became reactivated during the game when it was exposed to, and rehydrated by, the patient's sweat.

**Figure 4:**
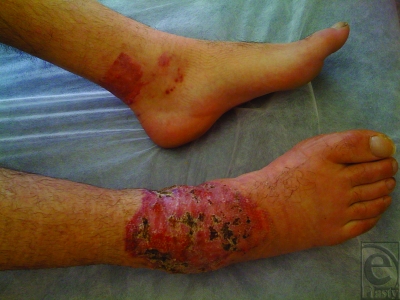
Healing wounds posttangential excision and split-thickness skin grafting.

The patient was managed initially with copious irrigation, and subsequently with tangential excision of the necrotic tissue and split-thickness skin grafting. He made an uneventful recovery and returned to playing football once his wounds had fully healed (Fig 4).

## DISCUSSION

Chemical burns may be caused by a range of substances and can present with various cutaneous and systemic manifestations. Thousands of potentially harmful chemicals are used everyday in industry and around the home, and include 4 basic groups: acids, alkalis, organic, and inorganic compounds. These all cause tissue damage by different mechanisms and therefore present with slightly different cutaneous signs; however, distinguishing this can be difficult at the time of presentation and so the ultimate management plan is not always straightforward.

Tissue damage, as a result of chemical contact, is determined by the following factors: concentration of the agent, quantity of the chemical, duration of contact, extent of penetration into the tissue, and its mechanism of action.^3^ Alkali burns are capable of deep tissue penetration and cause injury by liquefaction necrosis, which is saponification of fats and solubilization of proteins.^3^,^4^ Tissue destruction can continue long after the original exposure, commonly leading to an underestimate of the extent of the burn initially and delayed presentation.

Alkali burns are managed initially in the same way as for the majority of chemical burns—by removing any residual chemical and irrigating with copious volumes of water. This is often continued for a prolonged period of time while serial measurements of wound pH and body temperature are noted. Because of the deep tissue penetration of alkalis tangential excision and split-thickness skin grafting are commonly performed as a definitive procedure. Although these methods are widely accepted, literature exists that advocates neutralization of alkaline burns with solutions of a weak acid.^5^

The alkaline involved in this case was sodium hydroxide, a white, corrosive solid that is soluble in water, odorless, and soapy to feel.^4^ It is widely used in industry and in the manufacturing of drain and oven cleaners. Like other alkalis, it causes intercellular edema, erythema, decomposition of keratin, and destruction of the epidermis if not removed from the skin. Severe burns and deep ulceration can occur after several hours of exposure.^4^ Management is the same as for other alkalis.

## CONCLUSION

Alkali burns in association with sports have been well described in the literature, particularly those due to hydrated lime. This case highlights sports-related alkali burns with an unusual history, burns pattern, and mechanism of injury associated with a common household chemical.
